# The experience and discharge readiness of patients undergoing metabolic and bariatric surgery: a qualitative study

**DOI:** 10.3389/fmed.2025.1595361

**Published:** 2025-09-19

**Authors:** Shu Fang, Cheng Zhang, Meng Wei, Zhen Li, Jie Chen, Bilong Feng, Li Du

**Affiliations:** ^1^Department of Hepatobiliary and Pancreatic Surgery, Zhongnan Hospital of Wuhan University, Wuhan, China; ^2^Clinical Medicine Research Center for Minimally Invasive Procedure of Hepatobiliary & Pancreatic Diseases of Hubei Province, Wuhan, China; ^3^Department of nursing, Zhongnan Hospital of Wuhan University, Wuhan, China; ^4^College of Nursing, Florida State University, Tallahasee, FL, United States

**Keywords:** metabolic and bariatric surgery (MBS), discharge readiness, experience, qualitative study, nursing

## Abstract

**Background:**

The discharge readiness for metabolic and bariatric surgery (MBS) patients are designed to facilitate their transition from hospital to home and support early rehabilitation, ultimately improving discharge quality and outcomes.

**Objectives:**

This study aimed to describe the experiences and discharge readiness of patients undergoing MBS.

**Setting:**

A Tertiary hospital, China.

**Methods:**

An empirical phenomenological approach was used. Eighteen participants were recruited in Wuhan using convenience sampling. Semi-structured, in-depth, face-to-face interviews were conducted in May 2024. Each interview was transcribed verbatim, and Colaizzi’s method was used to analyze the data.

**Results:**

Five themes emerged from data analysis: post-surgical adaptation and expectations; negative emotions and stigma; knowledge gaps and ongoing education needs; post-discharge self-management and support needs; navigating physical recovery and long-term life planning. Patients faced both physiological and psychological discomfort. Insufficient social support was due to gaps in self-management knowledge and biased perceptions of reintegration. The surgery also impacted their reintegration into society and family life, while the long-term effect encouraged patients to adopt positive coping strategies and seek external support. Family provided primary support, but ongoing professional medical guidance was crucial for successful recovery and reintegration.

**Conclusion:**

The discharge readiness of the patient needs to be appreciated and improved. Developing a specific scale to assess readiness for MBS patients are crucial. A multidisciplinary, patient-centered discharge support system, supported by an information platform, should provide real-time, practical guidance for rehabilitation. Furthermore, establishing an effective post-discharge linkage mechanism will enhance self-management and ensure access to appropriate medical and social support.

## 1 Introduction

Obesity is a growing global health issue, with 2.5 billion adults (18 years and older) classified as overweight, and among these, 890 million were living with obesity and overweight in 2022 (1). The rising prevalence of obesity and overweight exerts a significant public health toll worldwide, contributing to 5 million deaths and 160 million disability-adjusted life-years in 2019 (2). Obesity is associated with health problems such as cardiovascular diseases, diabetes, and higher mortality (3). In China, the prevalence among adults are projected to reach 65.3%, with the medical expenses attributed to overweight and obesity potentially reaching 418 billion, accounting for about 21.5% of the total medical costs by 2030 (4). Metabolic and Bariatric Surgery (MBS) remains the most effective strategy for the treatment of obesity (5), which has been shown to result in sustained long-term weight loss, lower morbidity and mortality, and significant improvements in obesity-related comorbidities (6, 7). In China, the top three most performed MBS were sleeve gastrectomy (SG), one-anastomosis gastric bypass (OAGB), and Roux-en-Y gastric bypass (RYGB) (8).

Despite the literature demonstrating its efficacy, studies have shown that effective postoperative management of MBS is crucial for maintaining the effectiveness of surgery and preventing complications (9). With the development of enhanced recovery after surgery (ERAS), the length of stay in MBS has been reduced (10). Previous studies have shown that insufficient discharge preparation and ineffective postoperative management would lead to postoperative obesity, complications, and even increased readmission rates (11, 12). Recovery is a continuous process that extends beyond discharge. As patients recover from MBS, they may experience postoperative pain, nausea and vomiting, and tolerance of a complete liquid diet (13), and the patient’s feeling of fitness or willingness to be discharged (14). Therefore, discharge readiness is critical for patients to transition from hospital care to self-care at home, significantly impacts patients’ postoperative health behavior.

Discharge readiness refers to the comprehensive assessment by healthcare providers of the patient’s physiological, psychological, and social conditions, which are used to analyze and judge whether a patient can leave the hospital and return to family, society, and disease recovery (15). A previous study found that patients undergoing MBS under ERAS had a low level of discharge readiness, with scores averaging 7.45 ± 1.23, indicating a need for further improvement in discharge readiness at the center (16). The discharge readiness was influenced by various factors, categorized into four aspects based on Weiss ME and LB (17), namely, personal status (degree to which patients are physically ready to leave the hospital), perceived coping ability (degree to which patients feel that they can handle demands that they may face once home and take care of personal needs), knowledge (the degree to which patients are aware of the potential problems and restrictions that they could encounter after discharge), and expected support (the degree to which patients will have access to personal and medical assistance once home).

Owing to the significant changes imposed by MBS on individuals, including altered eating habits and physical appearance, people experience a period of physiological and psychosocial adjustment. Physical discomfort can increase anxiety at the time of discharge and make patients worry about not being able to cope when they get home (18). Many patients received negative comments from others due to undergoing bariatric surgery (19). These negative comments could affect patients’ social relationships and also require patients to adjust to new eating habits (20), which might not always be understood by others (21). The self-coping ability and health knowledge level of patients with MBS can promote the postoperative effect on recovery (22). The high level of discharge guidance can enhance patients’ confidence in treatment, reduce their uncertainty about treatment, and improve them self-care skills and knowledge of disease (23). Studies have shown that perceived family support positively impacts discharge readiness, with better social support correlating with higher readiness (24, 25).

Currently, the factors influencing the discharge readiness of bariatric surgery patients are in the preliminary research stage. Tools for assessing discharge readiness have been developed and validated in adult medical-surgical patients (17, 26). However, the readiness for hospital discharge scale (RHDS) has not been specifically tested in MBS populations. There is no specific tool to measure the discharge readiness of MBS patients and investigate the influencing factors. There is a lack of studies on the discharge readiness of patients is undergoing MBS.

Therefore, a qualitative study was conducted to analyze the experiences and the *status quo* of patients undergoing bariatric and metabolic surgery. Findings will help healthcare providers have a better comprehension of the *status quo* and existing problems regarding discharge readiness in MBS patients, assist them in better completing the transition from hospital care to return to society and family, and improve the discharge readiness and surgical effect of bariatric metabolic surgery on patients.

## 2 Materials and methods

### 2.1 Aims

This study aimed to describe the experience and understand the discharge readiness of patients undergoing bariatric and metabolic surgery.

### 2.2 Design

An empirical phenomenological approach was used to describe the experiences and understand the discharge readiness of patients who underwent MBS. This approach is widely used to explore individuals’ experiences of unique events people have encountered (27).

### 2.3 Sample

Participants who underwent MBS in the Obesity and Metabolic Disease Surgery Center of a Class Grade A hospital in Wuhan were recruited using convenience sampling in May 2024. Inclusion criteria were: (1) over 18 years of age; (2) meeting the surgical indications of the “Chinese Guidelines for Surgical Treatment of Obesity and Type 2 Diabetes (2019 Edition)” (28); (3) the MBS was performed for the first time in this center. Exclusion criteria were as follows: (1) participants with severe complications after MBS, e.g., (may give some examples of these complications); (2) history of mental illness or cognitive impairment; (3) have undergone revision bariatric surgery at this or other centers.

### 2.4 Data collection

Data were collected by the second author using semi-structured interviews focusing on participants’ experiences and the discharge readiness of patients. A semi-structured and in-depth interview was preceded by a face-to-face interview in a private meeting room. The aim of the research was explained, and informed consent was obtained from each participant before each interview. With participant permission, all interviews were audio-recorded and documented on paper. The interview content adhered to the interview outline in Table 1. During the interviews, appropriate techniques, such as questioning, probing, listening, paraphrasing, and responding, were utilized to encourage patients to express their opinions to the greatest extent possible and non-verbal cues such as tone, facial expressions, and body language of the interviewees should be meticulously paid attention to ensure the accuracy and comprehensiveness of interview information. Patients were encouraged to express them thoughts and experiences openly. Each interview session lasted approximately 40 min on average. At the conclusion of the interviews, the sample size was determined by the data saturation, and redundancy of themes emerged following 18 interviews.

**TABLE 1:** Interview guides.

No	Question
1	How are you physically prepared for discharge home, including wound healing, pain level, vomiting, flatulence, and any discomfort?
2	How are you mentally prepared for discharge home?
3	Did you experience mood swings after surgery? How do you deal with these emotions at the moment? Do you think you will encounter such problems after discharge?
4	Do you think you can take care of your daily life (e.g., grooming, eating, bathing, walking, going to the toilet) after discharge? What difficulties do you foresee? If not, can your family assist?
5	Did the nurse or case manager give you relevant health education (e.g., diet, medicine, health care products, exercise)? Do you think these are necessary for your discharge? Can this help you get ready for discharge? What do you think of the guidance for post-operative care?
6	After discharge, can you carry out self-rehabilitation and self-management at home according to the guidance, including drinking water, eating food and exercise? If not, why not? What help do you need?
7	Do you have confidence in self-management of diet, exercise, drugs and postoperative complications after returning home? If not, why? In which field?
8	After discharge, do you accept the potential impacts that MBS may have on your life and work? What are your primary concerns?
9	How do you plan your life and work arrangement after discharge? Do you need to make any necessary adjustments compared with pre-operation?
10	After discharge, if you encounter an emergency, how much help can you get? Who do you usually ask for help?
11	After discharge, what kind of support and help do you hope to receive from the people around you or medical institutions?
12	After discharge, how can you contact the medical team for necessary help and advice?

### 2.5 Ethical considerations

The studies involving patients were reviewed and approved by the Ethics Committee of Zhongnan Hospital Wuhan University (Approval number: 2021087K). Written informed consent was obtained from participants before the interview. Participation was voluntary and participants were informed that privacy and anonymity would be maintained.

### 2.6 Funding declaration

This work was supported by the Scientific and Technological Innovation Cultivation Fund of Zhongnan Hospital of Wuhan University (CXPY2020055) and Wuhan Nursing Association Youth Project (WHHL202421).

### 2.7 Data analysis

Audio recordings were converted to textual transcripts within 24 h after the interview completion. Interview data were then cross-verified against transcripts for refinement. In subsequent analyses, participant identifiers superseded real names to maintain confidentiality. Data were analyzed following Colaizzi’s method (29), which involved a meticulous review of the original data to extract phrases or sentences relevant to the themes. The analysis involved reading the transcript several times to achieve holistic comprehension, extracting significant statements or descriptive sentences, formulating and validating meanings through research team discussions to reach consensus, identifying and organizing themes into clusters and categories, and developing a thorough descriptive interpretation of the themes, describing the fundamental structure of the phenomenon, and finally validating the accuracy and comprehensiveness of the framework through feedback.

Several strategies were used to ensure trustworthiness and credibility. During the interview, in-depth interviews were conducted by a weight-loss case manager with rich clinical experience and systematic training in qualitative research. Two researchers analyzed the transcripts independently by bracketing data on preconceived ideas and strictly following the Colaizzi’s method described above. Findings were then compared and discussed by the team until consensus on themes, theme clusters, and categories was achieved. Meanwhile, all analytical steps and data were retained to ensure that every analytical step could be traced back to the original interviews.

## 3 Results

Eighteen participants, six of whom underwent Jejunojejunal bypass surgery (JJB) and 12 underwent Laparoscopic sleeve gastrectomy (LSG), were included in the study. Nine were females. Their ages ranged from 25 to 47 years. None had complications. Demographic characteristics of the participants are presented in Table 2.

**TABLE 2:** Demographic characteristics of patients undergoing metabolic and bariatric surgery (*N* = 18).

Participant	Gender	Age (years)	Highest level of education	Occupation/workplace	Marital status	Type of bariatric surgery	Complications	Comorbidities present	Primary caregiver
P1	Female	25	College degree	The fire safety monitoring center of the property management	Unmarried	JJB	None	Type 2 diabetes; Hyperlipidemia	Parents
P2	Female	38	Bachelor degree	Accountant	Married	LSG	None	Hyperuricemia	Husband
P3	Male	37	Technical secondary school	Electrical equipment company	Married	JJB	None	Hyperlipidemia;	Wife
P4	Male	28	Bachelor degree	Hospital logistics	Married	LSG	None	Hyperlipidemia; Hyperuricemia	Wife
P5	Male	30	Technical secondary school	Wuhan Iron and steel Company/now resigned for weight loss	Married	JJB	None	Hyperlipidemia; Hyperuricemia; Hypertension	Wife,Mother
P6	Female	23	Bachelor degree	Unemployed	Unmarried	LSG	None	Type 2 diabetes; Hyperlipidemia; Hyperuricemia	Mother
P7	Female	47	Vocational high school	Quality inspector in the workshop	Married	LSG	None	Type 2 diabetes; Hyperlipidemia	Husband
P8	Male	27	College degree	Insurance staff	Married	LSG	None	Hyperlipidemia; Hyperuricemia	Wife
P9	Female	31	College degree	HR manager	Married	LSG	None	Hyperuricemia	Husband
P10	Male	32	College degree	Other	Married	JJB	None	Hyperlipidemia; Hyperuricemia; Hypertension	Wife
P11	Male	26	College degree	Self-employed	Unmarried	LSG	None	Hyperuricemia	Parents
P12	Male	25	College degree	Freelancer	Unmarried	JJB	None	Type 2 diabetes; Hyperuricemia	Parents
P13	Female	28	College degree	Freelancer	Divorced	LSG	None	Hyperuricemia	Parents
P14	Male	25	Bachelor degree	Staff member	Unmarried	JJB	None	Type 2 diabetes; Hyperlipidemia; Hyperuricemia	Father
P15	Female	42	College degree	Self-employed	Married	LSG	None	Hyperlipidemia	Husband
P16	Female	30	Technical secondary school	Unemployed	Married	LSG	None	Type 2 diabetes; Hyperlipidemia; Hyperuricemia	Husband
P17	Male	36	High school	Driver/currently unemployed	Married	JJB	None	Type 2 diabetes; Hyperlipidemia; Hyperuricemia; Hypertension	Wife
P18	Female	34	Technical secondary school	Housewife	Married	LSG	None	None	Husband

JJB, jejunojejunal bypass; LSG, laparoscopic sleeve gastrectomy.

In this study, the patients’ experiences were reflected in five themes: (1) post-surgical adaptation and expectations; (2) negative emotions and stigma; (3) knowledge gaps and ongoing education needs; (4) post-discharge self-management and support needs; and (5) navigating physical recovery and long-term life planning. A consolidation of the themes describing patient experiences can be found in Table 3.

**TABLE 3:** The themes and subthemes of the discharge readiness of patients undergoing MBS.

Main themes	Subthemes
Post-surgical adaptation and expectations	1. Most patients adapted well physically and psychologically after surgery.
	2. Some patients experienced significant discomfort (pain, nausea, vomiting).
	3. Patients have high expectations for surgical outcomes.
	4. Patients with chronic conditions may not feel ready to discharge.
Negative emotions and stigma	1. Negative Emotions: Preoperative and postoperative examinations, anesthesia, and discomfort symptoms can trigger negative emotions, including tension, anxiety, and irritability.
	2. Stigma: Patients are sensitive to the topic of weight loss surgery and tend to avoid discussing it, especially with acquaintances.
Knowledge gaps and ongoing education needs	1. Knowledge of MBS needs to be improved and the complex nature of weight loss information requires ongoing support.
	2. Patients recognize the importance of health education; Patients claim the need for continuous professional guidance on diet, hydration, and exercise.
	3. Patients often have insufficient knowledge of self-management at home.
Post-discharge self-management and support needs	1. There are challenges in self-management after discharge, and ongoing professional guidance is needed.
	2. Family members play a crucial role in early home care but risk being inadequate.
	3. Healthcare professionals remain the preferred source for addressing health concerns, with patients valuing multiple channels of communication with healthcare providers.
Navigating physical recovery and long-term life planning	1. Short-term physical recovery after discharge affects work and daily life.
	2. Lack of effective self-management skills for home rehabilitation.
	3. Addressing home rehabilitation challenges requires effective and continuous social support to achieve optimal outcomes post-bariatric surgery.

### 3.1 Post-surgical adaptation and expectations

Most of the patients could adjust well to the physical and psychological changes after surgery and were ready for discharge, having a solid willingness to discharge. However, patients with discomfort symptoms, such as pain, nausea, and vomiting, poor tolerance of a complete liquid diet, and those with chronic diseases were not ready to leave the hospital.

#### 3.1.1 Most patients adapted well physically and psychologically after surgery

*There is no pain at the wound site, no vomiting, and the bloating has subsided (PN7)*

*I did not vomit, and then the wound is still (in) a little painful, but much better than yesterday, there was no flatulence. (PN9)*

*It uncomfortable now. Just a little uncomfortable on the day of surgery*…… *The recovery is getting better every day*……*After 48 h since yesterday, I was allowed to drink water, so I tried to drink one or two sips. When drinking, I felt my stomach hurt a little like being pulled, but then slowly I tried to drink another two sips and it got better. (PN4)*

*I don’t have too many mood swings, because my husband is very supportive of me. (PN5)*

*It was easy for me to get out of bed post-surgery. I was just vomiting. My wound is not painful. (PN13)*

*Acid reflux doesn’t affect discharge; I can tolerate it and should be fine with a few more days of rest at home. (PN14)*

#### 3.1.2 Some patients experienced significant discomfort (pain, nausea, vomiting)

With postoperative recovery, some patients still have varying degrees of physical discomfort.

*The bloating was terrible the first day after the operation, but today I’m much better with the gas. When coughing and moving, pulling to the abdominal wound, I still feel pain. (PN2)*

*My physical is not ready for discharge because of serious vomiting and weakness. (PN3)*

#### 3.1.3 Patients have high expectations for surgical outcomes

*I’m completely looking forward to going home. I feel strong enough to leave the hospital. Meanwhile, the physician has said to arrange for me to be discharged, I think I can leave the hospital. (PN5)*

*All the preparations have been prepared, and the protein powder nutrient solution have been purchased (PN2).*

*I’m ready, and I’m looking forward to getting out of here (hospital). (PN3)*

*I am looking forward to getting thinner after discharge. (PN7)*

#### 3.1.4 Patients with chronic conditions may not feel ready to discharge

Patients with insufficient postoperative physical preparation, such as obvious postoperative pain, significant gastrointestinal reaction, insufficient activity endurance, abnormal tolerance of a clear liquid diet, and preoperative chronic diseases and complications, were more worried about their health status and physical recovery after discharge. Discharge preparation was poor.

*My biggest concern is whether my blood sugar will return to normal after surgery. (PN8)*

*I just worried about the gout, nothing else very much. (PN10)*

### 3.2 Negative emotions and stigma

#### 3.2.1 Negative Emotions: Preoperative and postoperative examinations, anesthesia, and discomfort symptoms can trigger negative emotions, including tension, anxiety, and irritability

Some patients experienced certain negative emotions due to surgical stress or the operation itself. Due to the stress of surgery, patients felt tension and anxiety from the day of surgery to anesthesia. The discomfort of preoperative examination, postoperative pain, and discomfort also affected the patients’ psychological state, and even produced certain negative emotions.

*I was a bit panicky and anxious while waiting for the surgery, but after it was done, I was actually fine and felt relieved. (PN4)*

*The long waiting time for the inspection is an ordeal. The painless gastroscope was changed to an ordinary gastroscope, which made me feel painful and uncomfortable*…*when I can’t eat after surgery, having a sense of hunger and pain of gout, I feel a little irritable. (PN10)*

*When the vomiting is severe, there must be mood swings. I was pretty happy when I was admitted to the hospital and was looking forward to losing weight. I really regret it when I was not feeling well after surgery, and wonders if it’s painful to exercise to lose weight or if it’s painful now. But it’s okay to get through it now. (PN13)*

*After being discharged from the hospital, watching others eat will make me very envious because I can’t eat*…*But I think I shouldn’t have many negative feelings. (PN14)*

*Pain is not expected, so I have regrets (PN16)*

#### 3.2.2 Stigma: Patients are sensitive to the topic of weight loss surgery and tend to avoid discussing it, especially with acquaintances

*It depends on the relationship. Close friends who grew up together should be fine with it, but it might be considered taboo in the workplace. (PN1)*

*Maybe I’m still not that willing to tell others directly, especially people who are not close to me. (PN11)*

*I plan to meet them after I have lost weight. (PN6)*

Societal prejudice against individuals who were obese or have undergone MBS can cause patients to experience negative emotions, making them reluctant to disclose their surgery and seek social support.

*I’m afraid people who I am not close to will find out that I’ve had this surgery. I had the operation secretly from everyone. I’m an adult, and there’s no need to tell anyone what I do. I’ll take responsibility for my actions. I’ve been trying to lose weight for 12 years, and it’s made me depressed. Why can’t it be fixed and changed? (PN8)*

### 3.3 Knowledge gaps and ongoing education needs

The patients who returned to society and family after discharge were, to a certain degree, were affected by the surgery. The long-term effects of MBS require patients to cope positively and replan their future lives. However, the sudden change in their original habits can lead to had insufficient self-management ability after discharge, prompting them to seek help from others.

#### 3.3.1 Knowledge of MBS needs to be improved and the complex nature of weight loss information requires ongoing support

Due to poor postoperative physical conditions and excessive knowledge required for weight loss, patients often have a limited understanding of the MBS.

*I forget what the nurses said about MBS, I was asleep*… *I did listen to some of it, but maybe I wasn’t in the best state at that time. (PN13)*

*Yes, I understood it, but I’ve forgotten too much already. (PN16)*

For the patients, the knowledge of postoperative rehabilitation, such as postoperative nutrition absorption, wound care, complication identification, etc., was not well mastered and should be improved.

*My stomach is now shrinking, will it become larger in the future? Now that my stomach has shrunk, will I rely on the remaining stomach and small intestine to absorb nutrients in the future? (PN3)*

*I’m just worried it’s gonna hurt when I go back, and when are these scars gonna come off on their own, or am I gonna get rid of them? (PN15)*

The patients have a limited understanding of the types, symptoms, and precautions to avoid complications, and the importance of seeking medical attention in case of discomfort related to MBS. However, their overall understanding is not comprehensive, making it difficult to effectively identify complications.

*If I feel uncomfortable, I will definitely come to the hospital. I was just told some information, but I’m not very clear about the specifics since I haven’t experienced gastric fistula. (PN4)*

*The nurse told me that I might encounter hypoglycemia, so I should always carry some candies in my pocket. I forgot it (she mentioned). (PN5)*

#### 3.3.2 Patients recognize the importance of health education; Patients claim the need for continuous professional guidance on diet, hydration, and exercise

All patients affirmed the necessity of professional knowledge provided by healthcare providers during hospitalization. However, due to the complexity and abundance of information related to MBS, healthcare providers need to offer long-term professional support to assist patients in solving the various issues they face at different stages.

*Health education is very necessary because we have suddenly changed a lot in all aspects after the surgery. (PN15)*

*I need constant guidance to form a memory on my own. Despite having the brochures, I can’t always remember the information. I still hope that you will send me some information, e.g., some brief knowledge and short videos from time to time, which can be sent to the WeChat group, so that we can collect them and view them easily. (PN3)*

#### 3.3.3 Patients often have insufficient knowledge of self-management at home

Changes in dietary habits and adherence to a strict nutritional plan can affect the implementation of patients’ home diet rehabilitation. There may be misunderstandings of the knowledge during the implementation process. Healthcare providers need to provide continuous professional guidance to improve patients’ compliance.

*I think the duration of the liquid diet is a bit too long, isn’t it? (PN7)*

*I still have some pursuit for food. I’m sure I still want to eat normally cooked food. Just drink meal replacement powder, in fact, a liquid diet is not good for the recovery of gastrointestinal function, or there is a need for some food dross to allow the gastrointestinal movement. (PN4)*

*I may not eat exactly according to that chart. Sometimes I might make slight changes and make adjustments flexibly by myself. (PN18)*

### 3.4 Post-discharge self-management and support needs

#### 3.4.1 There are challenges in self-management after discharge, and ongoing professional guidance is needed

Patients face significant challenges in self-management after discharge, particularly in adhering to new dietary, hydration, and exercise regimens. They often struggle with long-term compliance and self-control, necessitating personalized plans and ongoing professional guidance.

*I was actually confused about what I should do all the time. I didn’t need to eat during my hospitalization because I was on nutritional fluids, but I don’t know what to eat when I go back*… *I will still ask you what I should eat at different stages. (PN15)*

Furthermore, patients initially accepted the different eating habits shortly after surgery, but doubted whether they could adhere to them for a long time.

*I will definitely try my best, at least strictly following the rules for the first and second months. Over time, I may become a bit lax, and perhaps when I can eat a wider variety of foods, I won’t follow it as strictly. (PN11)*

#### 3.4.2 Family members play a crucial role in early home care but risk being inadequate

Family members play a crucial role in providing support for daily tasks and self-care, especially during the initial recovery period. However, the social stigma surrounding obesity and weight loss surgery can make patients hesitant to seek broader social support.

*Family members can help change the dressing. (PN9)*

*My husband took photos of all the content on the notice board at the gate. After returning home, I don’t worry about my diet because my husband is very meticulous. (PN5)*

Societal prejudice against individuals who were obese or have undergone MBS can causes patients to experience negative emotions, making them reluctant to disclose their surgery and seek social support.

*I’m afraid people who I am not close to will find out that I’ve had this surgery. I had the operation secretly from everyone. I’m an adult, and there’s no need to tell anyone what I do. I’ll take responsibility for my actions. I’ve been trying to lose weight for 12 years, and it’s made me depressed. Why can’t it be fixed and changed? (PN8)*

#### 3.4.3 Healthcare professionals remain the preferred source for addressing health concerns, with patients valuing multiple channels of communication with healthcare providers.

*When I have a problem, my first instinct is to seek help from the doctor. seeking help from my family will not solve my problem. (PN4)*

*I seek help from my husband for the first time. We also have a WeChat group.if I have some unexpected situations, I will be the first time to reflect with you in the group. And I can go to the outpatient. (PN5)*

*Whenever I feel a bit unwell, I will contact you first. If the issue cannot be resolved, I will come to the hospital. (PN10)*

Furthermore, patients were aware of multiple ways to contact medical personnel to seek help.

*I have your WeChat group, a TikTok account, and the phone number from the official website (when I am not fine, I will contact you). (PN1)*

### 3.5 Navigating physical recovery and long-term life planning

#### 3.5.1 Short-term physical recovery after discharge affects work and daily life

The immediate post-discharge period is marked by insufficient physical recovery, which can significantly impact patients’ work and daily life, often requiring adjustments to their routines and responsibilities.

*I had planned to go to work on Sunday, but my strength didn’t allow me. I asked for another month off. According to the current state, I think it will have some impact on my work, and I am still worried about my physical strength. (PN3)*

*Because I have a baby, I’m afraid I don’t have the strength. (PN15)*

*I took 2 weeks off work for the surgery, and I only took 2 weeks off work. I’m busy with work. I’m gonna have to negotiate time off when I get back*…*I’m worried that I can’t keep up with my physical strength after surgery, and I’m afraid of delaying my work. (PN5)*

Some patients had insufficient self-care ability in the early period after returning home, and they will take the initiative to ask their families for help, especially for challenging behaviors such as dressing wounds, washing hair, bathing, and preparing meals.

*No one takes care of me, and I’m feeling a little overwhelmed. (PN2)*

*It might not be possible these days. I think I need help with walking. (PN16)*

*I can do daily life mostly by myself, but for things like preparing meals, I still need help from my family. (PN12)*

*I might need help changing the dressing on my wound. (PN9)*

#### 3.5.2 Lack of effective self-management skills for home rehabilitation

While patients generally have some plans for their future life post-surgery, they often lack the necessary self-management skills to implement these plans effectively. This gap highlights the need for continuous professional guidance to help patients navigate both their physical recovery and long-term lifestyle changes.

Before discharge, patients usually had certain arrangements and plans for their short-term, medium-term, and even long-term lives after returning home, which were mainly reflected in three aspects: diet, work and rest schedule, and exercise.

*First, I have to change my daily routine, because it was so irregular before. Second, according to the requirements of the brochure on the diet, I will follow the diet plan after discharge*…*I want to see if I can adapt to it. Third, I can go out for a walk after dinner every day, and also cooperate with some appropriate exercise*… *These are the main aspects. (PN1)*

*If my gout subsides and I’m better, I will firstly try basic exercise, like walking slowly after eating. After 3 or5 months, I will try to climb the stairs and do aerobic exercise. I also plan to get a girlfriend. (PN15)*

Most patients face a significant dilemma, and their coping ability in self-management was insufficient after discharge. Although they were aware of the necessity and importance of diet, drinking water, and activities as prescribed by the healthcare providers, they were still worried about whether they could adhere to the prescribed plan. This led patients to present a mixed attitude during the interviews: on the one hand, they believed they could adhere to a regulated lifestyle, but on the other hand, they were very concerned about their lack of self-control.

*The biggest problem for me should be the self-control of my diet. In fact, if I had strong self-control, I basically would not need to do this surgery. It’s often because of inadequate self-control that people resort to surgery, right? (PN1)*

*I think the diet will be a big test for people like us who love to eat. It’s a matter of self-control. My husband is a great cook, so I’ll try to avoid the dining table during mealtimes. (PN5)*

*I might be a little worried about whether I can stick to a diet at home. (PN11)*

*To be honest, it’s too hard for me to keep exercising. I’ve tried to lose weight through exercise for 3–4 months before the surgery*…*Although it was effective, I didn’t stick with it. (PN10)*

#### 3.5.3 Addressing home rehabilitation challenges requires effective and continuous social support to achieve optimal outcomes post-bariatric surgery

The transition back to society involves not only managing physical limitations but also developing strategies for sustainable lifestyle modifications, emphasizing the importance of ongoing support from both healthcare professionals and family members in achieving long-term success.

Patients re-entering society often experience cognitive biases that lead to inadequate social support. They may have an inaccurate perception of their health condition, resulting in misunderstandings about the support they need and difficulties in expressing their needs effectively.

*Actually, I think it’s a little early to talk about this. It is only when problems are encountered that it is more specific. Now I don’t really know what to say (and I don’t also know how to solve the problem). (PN1)*

*I don’t know what I can do now. I think I will have to wait until it happens. (PN2)*

*In the later stages, such as specific phases, if I’m unsure about what to do, I might ask you about dietary recommendations for that stage. Otherwise, there won’t be much else to address. (PN15)*

## 4 Discussion

This study was designed to systematically characterize the lived experiences and discharge readiness of patients undergoing MBS. Through an in-depth exploration of these dimensions, the findings offer evidence-based guidance for optimizing discharge preparation protocols and improving postoperative recovery trajectories in this patient population.

### 4.1 Fully assessing the discharge needs of patients and formulating personalized discharge preparation service plans

Patients’ perception of discharge readiness was an important aspect in evaluating their readiness for discharge (17). Patients may experience some discomfort after surgery, but these symptoms would gradually improve over time. It is important to emphasize that early postoperative discomfort is a normal part of the body’s adjustment process, and the gradual improvement of symptoms is a typical indication of recovery. Although some patients experienced vomiting, pain, and other discomforts that might affect their readiness for discharge, this study found a strong expectation for discharge and optimism about weight loss outcomes. This may be due to advancements in the surgery and the promotion of the ERAS, which enables patients to achieve better surgical outcomes and experience fewer complications, thus facilitating faster postoperative recovery (30). Before choosing to undergo surgery, patients typically go through an extensive decision-making process. By researching the recovery experiences, lifestyle changes, and complications faced by others who have had the same surgery, they improve their ability to manage similar issues post-surgery, which fosters a more positive attitude toward home recovery after discharge. However, patients’ high expectations for surgical outcomes may influence their discharge decisions and postoperative psychological state (31). Healthcare providers should conduct a thorough assessment of patients based on their performance during previous treatments. A discharge preparation plan should be tailored to each patient’s individual needs, with both short-term and long-term goals set. Enhancing the patient’s proactive role in self-rehabilitation while working toward these goals can minimize the risk of disappointment due to mismatches between expectations and actual results. In addition, this study indicated that patients experiencing severe symptoms, such as vomiting, pain, and other discomforts, as well as those with existing complications, were often more concerned about their recovery after discharge and typically had lower levels of discharge preparedness, which was consistent with previous studies (12, 32). The research showed that symptom management predicted increased patient readiness for hospital discharge (33). Thus, healthcare providers should accurately and comprehensively assess patients’ discharge preparation needs, correctly handle physical discomfort symptoms, and provide personalized guidance, especially for those with complications, to alleviate their physical discomfort and enhance their readiness for discharge.

### 4.2 Addressing patients’ psychological needs and facilitating their post-discharge recovery

This study found that patients undergoing MBS experienced negative emotions during the perioperative period due to surgical stress, discomfort from examinations, changes in dietary habits, and recovery status, which was consistent with previous research (34). Patients with higher pre-surgery anxiety often faced more significant challenges adjusting and recovering post-surgery (35). Therefore, medical personnel should pay attention to patients’ psychological changes, analyze the causes of their negative emotions, and address the stressors contributing to these feelings, and encourage patients to actively discuss their concerns with family and friends to alleviate negative feelings. Additionally, patients should be guided on effective ways to express and manage their negative feelings. Furthermore, this study confirmed that obese individuals often experience social stigma during the recovery process, which was consistent with previous findings (19). Such stigma can lead to weight-related embarrassment, prompting patients to use avoidance strategies when discussing their weight and surgery (36). Patients undergoing MBS who have faced discrimination or social exclusion due to obesity may develop anticipated stigma, leading to social withdrawal (37). Puhl and Heuer (38) found that obesity-related stigma can influence the healthcare-seeking behavior of individuals with obesity, which can subsequently affect their postoperative recovery. Therefore, healthcare providers should focus on educating and supporting patients’ mental health to meet the patients’ postoperative rehabilitation needs when formulating the home self-management plan for discharge preparation. On the one hand, studies have shown that family members play an important role in reducing obesity-related stigma (39); thus, effectively reducing stigma among relatives and friends significantly benefits patient recovery. On the other hand, social media should standardize the coverage of people with weight loss, avoid the stereotype of obesity, improve the public’s prejudice against obesity, and create a respectful and fair social environment, reduce the stigma of patients, and help them achieve social reintegration as soon as possible.

### 4.3 Enhancing patients’ professional knowledge for self-management at home and providing sustainability professional guidance

Professional knowledge is crucial for patients undergoing MBS. However, studies indicate that 30%–50% of these patients have low levels of health literacy (40). Many reasons could affect patients’ health knowledge, including the complexity of knowledge, postoperative discomfort affecting the mastery of professional knowledge (22), and the level of guidance nurses provide (33). Discharge instructions can be confusing for patients with low levels of health literacy. Notably, our study found that all patients recognized the significance and necessity of the professional knowledge provided by healthcare providers, aligning with previous research (41). Therefore, healthcare providers need to offer health education after completing professional knowledge training on weight loss. It’s also important to assess the patient’s capacity for information reception and select appropriate timing to avoid discomfort that might hinder their understanding. Additionally, our study revealed that patients rely on their families during home rehabilitation. Allowing the family members to participate in this process can enhance the effectiveness of the information acquisition (42). So, Improving the health knowledge of patients’ families can significantly enhance patients’ readiness for discharge.

Given that the length of stay for bariatric patients has been reduced under the Enhanced Recovery After Surgery (ERAS) protocols, patients are primarily undergoing home-based rehabilitation. Patients and their family members often perceive that they are not adequately prepared for discharge and have attributed post-discharge problems to their unmet information needs (43). Therefore, there is a need to build an information management platform. Studies have shown that patients’ compliance with home-based rehabilitation has been improved through the use of information management platforms for health knowledge inquiries and continuous professional guidance (44). At the same time, nursing case managers can use this platform to provide information consultation and assistance for home rehabilitation. The nurse case managers have proven beneficial in managing health conditions, facilitating self-management, reducing hospitalizations, and improving quality of life (45). Healthcare providers can enhance the accessibility and perception of information by using multimedia support. This allows patients to learn repeatedly in a convenient manner, achieving the desired effect of professional guidance (46). Additionally, the nursing case managers utilize online platforms such as telephone follow-ups and information management applications to optimize communication with patients, assess their understanding, and identify knowledge gaps to tailor targeted health education plans. Continuous and personalized health education can meet the information needs of different patients, helping them establish long-term self-management awareness. This process ultimately facilitates the maintenance of healthy behaviors among postoperative individuals (47, 48).

### 4.4 Making full use of social support to promote home-based rehabilitation

Although most patients in this study expressed a willingness to seek help, they often had unclear understanding of their own needs and may have lacked sufficient social support. Several factors contribute to this phenomenon. Firstly, patients may have experienced discrimination or social exclusion due to obesity, leading to anticipated stigma and social withdrawal, which hinders their willingness to seek help from others (36). Secondly, patients may have an inadequate understanding of their self-management abilities, resulting in deviations in their expectations and actions. This study has shown that family members are the primary source of help for patients, providing emotional support and life care, which was consistent with Busebaia et al. (49). However, the home life of patients after MBS has changed significantly compared to their pre-MBS life, and families may struggle to adapt to these new changes, resulting in low support levels. Weak family social support would create a poor rehabilitation environment and increased resistance to patients’ self-management efforts (50). While family support is crucial, it is often limited. The level of support received from family members, friends, and healthcare providers upon being discharged can significantly affect patients’ behavioral modifications and surgical outcomes (42). Therefore, in addition to providing health education to patients, healthcare providers should strengthen communication with their families, encourage family members to be involved in patients’ home self-management, and guide both patients and their family members to adapt to new changes. This collaborative effort can create a positive atmosphere for self-management at home (49). Moreover, healthcare providers can offer patients more opportunities to obtain peer support. Studies have shown that peer support plays an important role in influencing MBS-related outcomes or experiences, as individuals with similar experiences are more likely to empathize, encourage, and help each other (51). Therefore, effectively utilizing social support can meet patients’ rehabilitation needs after discharge.

### 4.5 Improving patient readiness for discharge and increasing patients’ coping ability

Our study found that short-term self-recovery after discharge was inadequate, adversely affecting patients’ daily life and work, consistent with earlier studies (12, 32). Patients reported feeling unable to manage self-care tasks shortly after discharge and needed assistance from family members for activities such as bathing and changing wound dressings. Additionally, due to postoperative diet restrictions and fatigue, patients require a period of rest at home before returning to their normal activities. Therefore, patients need to seek support from family or medical institutions to effectively manage self-care and coping tasks (52). A study has demonstrated that 20%–30% of patients experienced varying degrees of weight regain within 2 – 10 years post-surgery (53). Poor post-discharge self-management, including irregular diet and follow-up, could contribute to the recurrence of obesity (54). Galvin et al., (55) suggested that readiness for hospital discharge could improve patients’ competence to manage self-care at home and receive adequate support to cope with various demands post-hospitalization. By understanding patients’ discharge preparation needs, personalized guidance can be provided to enhance their readiness and coping abilities. Our study revealed that while most patients were able to develop self-management plans for a healthier lifestyle post-discharge, they struggled with long-term self-management, a finding consistent with other studies (48, 56). Therefore, healthcare providers should offer personalized information and guidance tailored to each patient’s unique lifestyle to meet their individual needs and improve compliance (57).

Furthermore, our study found that patients have recognized the impact of being overweight on their work and believed that achieving a healthier weight would improve their employment opportunities. Hence, patients should be encouraged to manage the transition to postoperative recovery effectively, build positive social relationships, and strengthen their self-confidence in self-management. By establishing and enhancing patients’ self-confidence in self-management, cultivating the spiritual motivation to adopt healthy living habits, and improving their self-management after MBS, lasting changes in behavior can be promoted (58).

## 5 Limitations

This study has several limitations. Because of shame, participants might have been reluctant to describe their experiences and emotions fully, and they may be worried about the confidentiality of their responses. To address this, a paper informed consent form was signed before each interview, the aim of the research was explained, and every participant was informed that the interview would be analyzed securely and confidentially. Secondly, the physical discomfort experienced by patients would affect the interview results. Additionally, this study was conducted from a qualitative perspective. Including quantitative evaluation indicators could provide a more comprehensive analysis. Lastly, participants were recruited from only one hospital, which may not accurately reflect the experiences and discharge readiness of the patients undergoing MBS from other hospitals or regions in China.

## 6 Conclusion

This study explores the experiences and discharge readiness of patients undergoing MBS. It reveals a need to enhance patients’ discharge readiness and emphasizes the importance of continuous and effective medical professional support to ensure smooth recovery post-surgery and discharge. This study comprehensively analyzes the discharge readiness of patients from multiple perspectives, highlighting the necessity for a specific assessment scale tailored for MBS patients. Key findings and recommendations emerged. Firstly, a discharge preparation service intervention program should be developed. This program, initiated early in the hospital stay and led by healthcare providers or case managers in collaboration with the surgical team, must proactively address patient mood and systematically build essential post-discharge self-management competencies (e.g., wound care, dietary adherence, activity progression, medication management, and complication recognition). Secondly, continuous professional guidance is crucial for patients transitioning from hospital to home, ensuring they receive the necessary guidance for effective rehabilitation. Form a core team responsible for post-discharge guidance, including the bariatric surgeon, primary nurse, registered dietitian, physical/occupational therapist, mental health professional, and a case manager, whose duties are to develop and implement multidisciplinary, patient-oriented discharge preparation support service. An integrated information system designed to provide real-time, scientific, and effective rehabilitation guidance for patients at home should be built. Specifically, the system deploys an information platform featuring: a patient portal, remote monitoring capabilities, automated symptom assessment tools, and secure communication channels. Furthermore, a real-time data-driven proactive intervention and tiered alert system is established. This comprehensive framework dynamically delivers evidence-based guidance and automated referral pathways, ensuring the safety and scientific rigor of home-based recovery and ultimately enhancing long-term outcomes. Finally, an effective linkage mechanism should be built. A robust linkage mechanism post-discharge can enhance patients’ self-management abilities and increase access to appropriate medical or social support, thereby ensuring successful postoperative weight loss and home safety. By implementing these recommendations, the discharge readiness of MBS patients can be significantly improved, facilitating smoother recovery and better long-term outcomes.

## Data Availability

The original contributions presented in the study are included in the article/supplementary material, further inquiries can be directed to the corresponding authors.
